# Influence of Ulluco Starch Modified by Annealing on the Physicochemical Properties of Biodegradable Films

**DOI:** 10.3390/polym14204251

**Published:** 2022-10-11

**Authors:** Luis Daniel Daza, Daniela O. Parra, Carmen Rosselló, Walter Murillo Arango, Valeria Soledad Eim, Henry Alexander Váquiro

**Affiliations:** 1 Department of Chemistry, University of the Balearic Islands, Ctra Valldemossa, km 7.5, 07122 Palma de Mallorca, Baleares, Spain; 2 Departamento de Producción y Sanidad Vegetal, Facultad Ingeniería Agronómica, Universidad del Tolima, Ibagué 730006, Colombia; 3 Departamento de Química, Facultad de Ciencias, Universidad del Tolima, Ibagué 730006, Colombia

**Keywords:** *Ullucus tuberosus* Caldas, biopolymer, biodegradable films, packaging, starch modification

## Abstract

This work aimed to evaluate the use of annealing (ANN) ulluco starch in the preparation of biodegradable films and its impact on the physicochemical properties of the materials. Three film samples (FS1, FS2, and FS3) were prepared at a fixed starch concentration (2.6% *w*/*v*) using glycerol as a plasticizer and then compared to a control sample (FSC) prepared with native ulluco starch. The physical, mechanical, and thermal properties of the films were evaluated. The use of ANN starch decreased the solubility (from 21.8% to 19.5%) and the swelling power (from 299% to 153%) of the film samples. In addition, an increase in opacity and relative crystallinity (from 7.54% to 10.5%) were observed. Regarding the thermal properties, all the samples presented high stability to degradation, with degradation temperatures above 200 °C. However, the samples showed deficiencies in their morphology, which affected the barrier properties. The use of ANN starch has some advantages over native starch in preparing films. However, more analysis is needed to improve the barrier properties of the materials. This work reveals the potential of the ANN ulluco starch for biodegradable film preparation. In addition, the use of modified ulluco starch is an alternative to add value to the crop, as well as to replace non-biodegradable materials used in the preparation of packaging.

## 1. Introduction

Plastics are inexpensive materials with exceptional characteristics, such as great strength, versatility, water impermeability, low conductivity, corrosion resistance, and durability, making them an essential raw material for different industries. As a consequence of its industrial importance, in 2020, the worldwide production of plastic reached 367 million metric tons [1]. Production is expected to increase twofold by 2040 [2]. However, plastics are non-biodegradable materials synthesized from non-renewable resources. Their post-consumer disposal, mainly of single-use plastics, can cause air, water, and soil pollution, severely impacting the environment and human health [3]. Consequently, different policies and strategies have been implemented to reduce their environmental impact [4]. Among these strategies, the search for biodegradable and renewable materials that can be used to prepare alternative substitutes for plastic has become highly relevant [5].

Biodegradable packaging materials are sustainable and eco-friendly materials based on synthetic (polylactic acid (PLA), polyvinyl alcohol (PVA), and polycaprolactone (PCL)) or natural (proteins, polysaccharides, and lipids) polymers, which can serve as substitutes for non-biodegradable plastics [3]. Among the natural polymers, starch is one of the most widely used in preparing biodegradable packaging due to its abundance, biodegradability, low cost, and renewability. Different studies have evaluated the potential of starches obtained from non-conventional sources to produce biodegradable packaging [6] to ensure food security and generate added value to underexploited crops. Although starch-based films are characterized by low oxygen permeability, they have disadvantages such as poor mechanical properties and higher solubility. Starch can be enzymatically, chemically, or physically modified to overcome these drawbacks. Annealing (ANN) is a physical modification in which a reorganization of starch granules occurs at temperatures between the glass transition (*Tg*) and the onset of gelatinization (*To*) and with an excess of moisture [7]. The main effects of annealing on the material are increased crystallinity, thermal stability, granule size and molecular mobility, and reduced water absorption capacity [8].

In recent years, starch obtained from ulluco (*Ullucus tuberosus*), an Andean tuber, has gained attention due to its high extraction yield [43–65% dry basis (d.b.)] along with its amylose content (between 28% and 36% d.b.), which makes it suitable for use in the production of biodegradable packaging due to the high transparency, excellent stability against temperature, and good texture and water vapor permeability characteristics [9,10]. Additionally, ulluco starch has shown excellent compatibility with chitosan to produce biodegradable packaging [11].

Ulluco starch was even successfully tested as a coating for goldenberries, increasing the shelf life of the fruits [9]. More recently, ANN modification of ulluco starch has been shown to improve the physicochemical characteristics of native starch. However, there is no information related to the use of modified ulluco starch in the preparation of biodegradable packaging. Based on the above considerations, this work compares the physicochemical properties of biodegradable films prepared with native ulluco starch and ulluco starch modified by ANN.

## 2. Materials and Methods

**Figure 1:**
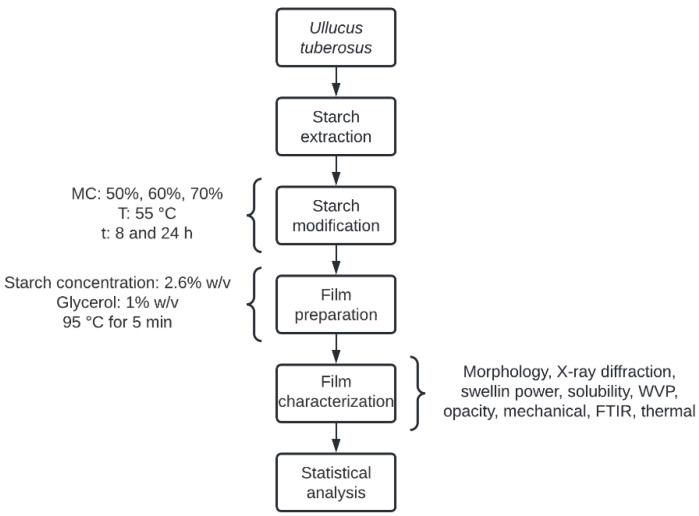
Methodology flowchart.

The methodology of this work is summarized in Figure 1.

### 2.1. Raw Material and Starch Extraction

Ulluco tubers (*Ullucus tuberosus* Caldas) were acquired at a local market in Ibagué (Colombia). The native starch (amylose content 27.9%) and the annealing modified starch (ANNS) were obtained according to the methodology described by Valcárcel-Yamani [12] and modified by Parra et al. [7]. In brief, after adjusting the moisture content (MC) at 50% (S1), 60% (S2), and 70% (S3), the starch was heated at 55 °C for 8 h (S1) or 24 h (S2 and S3). Then, the samples were dried at 35 °C until reaching a moisture content less than or equal to 10%. Starches were ground with a mortar and stored at room temperature (~25 °C). Three annealed starches were obtained ANNS1 (50% MC and 8 h at 55 °C), ANNS2 (60% MC and 24 h at 55 °C), and ANNS3 (70% MC and 24 h at 55 °C). The treatments were selected considering the best physicochemical properties of modified starch to obtain biodegradable films. These properties were evaluated and discussed in a previous study [7].

### 2.2. Film Preparation

The films were prepared using the casting method following the methodology described by [10]. Native ulluco starch, ANNS1, ANNS2, and ANNS3 were dissolved in distilled water, at a concentration of 2.6% (*w*/*v*), with magnetic stirring for 5 min. The mixture was heated to 95 °C and maintained at this temperature for 5 min. After that, the mixture was cooled to 50 °C, and glycerol was added at a concentration of 1.0% (*w*/*v*). The solutions were stirred for 10 min and then transferred to a Petri dish with a diameter of 8.5 cm (~0.53 g of the film solution per cm^2^). The films were dried at 40 °C for 24 h. After the drying process, native ulluco starch film (FSC), ANNS1 film (FS1), ANNS2 film (FS2), and ANNS3 film (FS3) were obtained and stabilized at a relative humidity (RH) of 57 ± 3% and a room temperature (~25 ± 2 °C) for at least 24 h, before the analyses.

### 2.3. Morphology of Edible Films

Micrographs of the surface of the samples were acquired using scanning electron microscopy (S–3400N, Hitachi, Düsseldorf, Germany) at an accelerating voltage of 15 kV under vacuum pressure of 40 Pa and different magnifications. For the analysis, samples were placed on stubs using double-sided carbon tape and covered with a gold layer.

### 2.4. X-ray Diffraction (XRD) of Edible Films

X-ray diffraction patterns were analyzed using a Bruker D8 Advance diffractometer (D8 advance, Bruker, San Jose, CA, USA) operating with Nickel filtered and Cu-Kα1 radiation, 40 kV and 40 mA. The wavelength used was 1.5406 Å. Data collection was carried out in the 2θ range of 3.5–70°, with a step size of 0.02035° (2θ) and a counting time of 0.8 s/step. Relative crystallinity was determined after deconvolution of peaks from diffractograms by the relationship between the crystalline peak area (***Ac***) and the total area (***At***) using Equation (1) [13]. Results were reported as the average of three measurements.

(1) $$Relative\;crystallinity\;\left(\%\right)=Ac/At\times 100$$

### 2.5. Swelling Power and Solubility

Swelling power (SP) and solubility (S) were calculated using the methodology reported by Baron et al. [14] with some modifications. Films specimens (2 cm × 2 cm) were weighed within pre-weighed aluminum capsules. Subsequently, the pieces were dried at 110 °C for 24 h and weighed (w1). The dried films were placed in glass bottles containing 50 mL of distilled water and stored for 24 h at room temperature (~25 ± 2 °C). Then, excess water was gently removed from the surface of the samples, and the mass gain of the swollen films was measured by weighing (w2). Finally, the pieces were dried at 110 °C for 24 h, and the final weight was recorded (w3). The moisture content (MC, %), swelling power (SP, %), and solubility in water (S, %) of the edible films were calculated using Equations (2) and (3), respectively.

(2) $$SP=\left[\left(w_{2}-w_{1}\right)/w_{1}\right]\times 100$$

(3) $$S=\left[\left(w_{1}-w_{3}\right)/w_{1}\right]\times 100$$

### 2.6. Water Vapor Permeability

The water vapor permeability (WVP) was determined according to [15]. Results were reported as the mean of four repetitions.

### 2.7. Opacity

The absorbance of US edible films was measured at 600 nm using an ultraviolet–visible spectrophotometer (Genesys 10S UV-VIS, Thermo Scientific, Thermo Fisher Scientific, Waltham, MA, USA). The OP was calculated using Equation (4).

(4) $$OP=Abs_{600}/FT$$

where $Abs_{600}$ is the absorbance at 600 nm and FT is the film thickness (mm). Results were reported as the average of twelve repetitions.

### 2.8. Mechanical Properties

Samples with dimensions of 5.0 × 1.0 cm were loaded in a texturometer (LS1, Lloyd Ltd., Largo, FL, USA) and their mechanical properties were evaluated according to [16]. The crosshead speed and initial distance were 5 mm/min and 4 cm, respectively. The tensile strength (TS, MPa), Young modulus (YM, MPa), and elongation at break (EB, %) were calculated using the Nexygen Plus software (Lloyd ltd., Version 3.0). Results were reported as the average of eight measurements.

### 2.9. Attenuated Total Reflectance-Fourier Transform Infrared Spectroscopy (ATR-FTIR)

The Fourier-transform infrared spectroscopy (ATR-FTIR) spectra of samples were determined using a Fourier transform infrared spectroscopy (Tensor 27, Bruker Optics, Ettlingen, Germany) equipped with the Platinum ATR accessory (Bruker). The spectra were obtained with a resolution of 4 cm^−1^ and 40 scans over the range of 4000–700 cm^−1^.

### 2.10. Thermal Analysis of Starch and Edible Films

The weight loss (WL) and thermal degradation temperature (TD) of film samples were assessed using a thermogravimetric analyzer (SDT 2960, TA Instruments, Tokyo, Japan) according to the method described by [11]. A temperature range between 30 °C and 600 °C was used, with a heating rate of 10 °C, under a nitrogen stream.

The dynamic-mechanical thermal behavior of film samples was evaluated using a dynamic mechanical analyzer (DMA, Q800, TA Instruments, New Castle, DE, USA) following the method described by [17] with some modifications. Films specimens (30 mm × 9 mm) were loaded and submitted to a range of temperatures between −80 °C and 150 °C with a heating rate of 10 °C, at a constant frequency of 1 Hz and strain amplitude of 10 μm. For thermal analysis, the result was reported as the mean of three repetitions.

### 2.11. Statistical Analysis

The mean and standard deviation of the results for at least three replicates under each treatment were reported. Analysis of variance (ANOVA) at the 95% confidence level was used to determine any statistically significant differences. Multiple range tests (MRT), using the Tukey–Kramer range test, were used to determine which mean results differed significantly from others. ANOVA and MRT were performed using MATLAB^®^ software (Version R2019b, The MathWorks Inc., Natick, MA, USA).

## 3. Results

### 3.1. Morphology of Biodegradable Films

**Figure 2:**
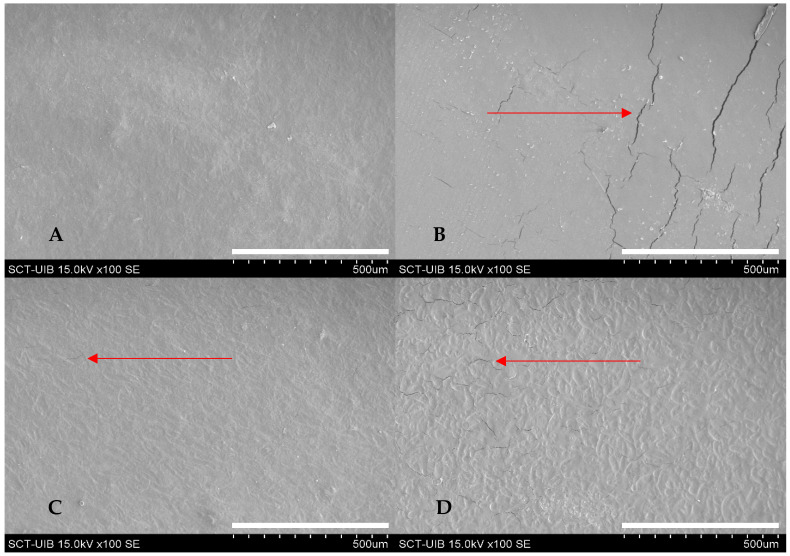
SEM micrograph of FSC (**A**), FS1 (**B**), FS2 (**C**), and FS3 (**D**) at magnification of 100×.

Micrographs of the films evaluated are shown in Figure 2. In general, all the samples presented roughness on the surface. Only FSC showed a continuous morphology without cracks or porosities. In contrast, the FS1 and FS3 samples showed macrocracks on their surface. However, FS2 shows only some minor cracks (microcracks). These defects may be due to shrinkage of the starch phase after drying and may negatively affect material properties such as water vapor permeability and mechanical properties. A similar phenomenon was observed in hydroxypropyl methylcellulose/hydroxypropyl starch films [18]; the authors related the presence of the micro and macrocracks on the films’ surface with the decrease in elongation and increase of gas permeability. These micro and macro fractures may also be associated with the high degree of ordering of the modified starch compared to the native starch. This organization can reduce the interaction between the polymer chains and the plasticizer (glycerol), which would generate more fragile and brittle films, which agrees with previous reports [19]. A possible strategy to mitigate the appearance of these fractures in films prepared with modified starch may involve using higher plasticizer concentrations or a longer heating time at 95 °C.

### 3.2. X-ray Diffraction (XRD) of Edible Films

**Figure 3:**
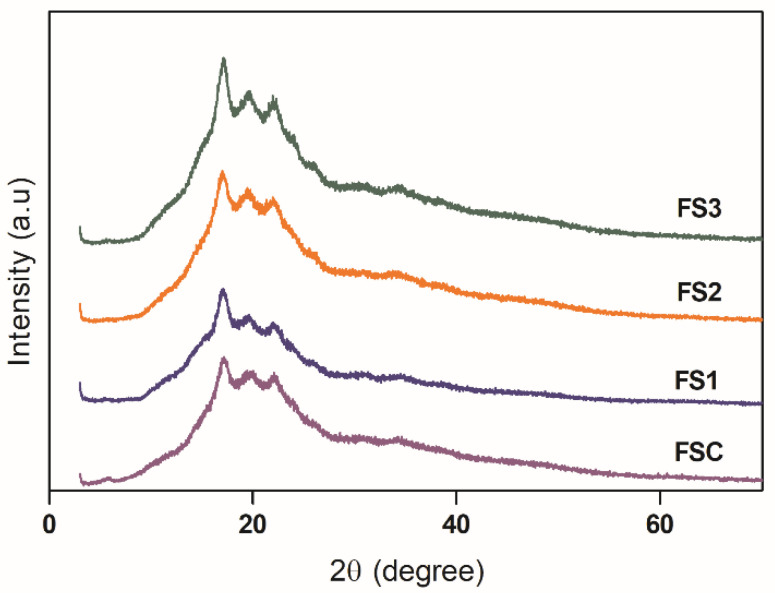
XRD diffractograms of native and modified starch-based films samples.

The XRD patterns of biodegradable films are presented in Figure 3. All samples showed a typical B-type crystal structure similar to the native ulluco starch [7]. This structure showed characteristic peaks at around 5.6° 2θ and 17° 2θ (peak with strong diffraction intensity) and with small peaks at around 15°, 20°, 22°, and 24° 2θ. The crystalline regions in the evaluated films are characteristic of semicrystalline materials and agree with the results obtained for guinea arrowroot starch-based films [20]. In addition, relative crystallinity of the biodegradable films was 7.5 ± 0.8%, 10.5 ± 1.2%, 8.03 ± 0.19% and 8.5 ± 0.8% for FSC, FS1, FS2, and FS3, respectively, with statistical differences between FS1 and the other samples (*p* < 0.05). The difference between the relative crystallinity values of the films can be associated with the reorganization of the starch granule structure in the modification process [8]. Other researchers have also observed the recrystallization process in starch films stored under different temperature and relative humidity conditions. They have associated this behavior with the process of retrogradation and realignment of the polymer chains, which depends on factors such as the amylose/amylopectin ratio, the presence and concentration of plasticizers, and the glass transition temperature [19]. High relative crystallinity of the materials is associated with a low permeability to gases, which is due to a greater organization of the polymer chains that will prevent the passage of water molecules. However, as mentioned above, the presence of micro and macrocracks on the surface of the films increases their passage.

### 3.3. Solubility and Swelling Power of Biodegradable Films

The solubility and swelling ability of the films obtained with annealed starch were lower than the FSC (Table 1). However, only samples FS2 and FS3 presented statistical differences for solubility compared to FSC (*p* < 0.05). The decrease in these parameters may be because the ANN process produces a reorganization of the molecular structures in the starch granule, strengthening the interactions between amylose and amylopectin, thus generating a more stable structure, or increasing the crystallinity of the starch [8]. Since solubility is related to the affinity of the materials to water, a low value of this parameter could be related to a better resistance or structural integrity of the films. The solubility values obtained in this work were similar to those reported for films obtained using heat-moisture treatment (HMT) on modified rice starch [21] and potato starch [22] but lower than those reported for films based on modified pearl millet starch [23]. Despite having been subjected to thermal modification, starches may present differences in their physicochemical properties due to differences in the botanical source of the material, the amylose content, and the parameters of the modification process.

The FSC showed the highest swelling power (*p* < 0.05), followed by FS2, FS1, and FS3. A decrease in the swelling power was observed in the films prepared with ANN starch compared to FSC. Such decreases in swelling power in films prepared with modified starch are related to the reduction of free volume in the film structure, which is a consequence of the crystallinity increase [24]. Although a reduction in the swelling power of the films obtained with modified starch was observed, the values obtained in this work were higher than those reported for films based on acha (93.1%) and iburu (99.5%) starch [25]. As mentioned above, the differences in the physicochemical properties between films obtained with different starches may be due to the type of starch used (native or modified), botanical source, amylose content, and crystallinity.

### 3.4. Water Vapor Permeability of the Films

The purpose of food packaging materials is to isolate the food matrix from environmental factors such as light, oxygen exposure, and water vapor, thus increasing its shelf life. The permeability of a film is defined as the amount of water vapor that passes through a unit area of the material per unit of time [9]. The WVP values of biodegradable films are shown in Table 1. The highest permeability was observed in films FS1 and FS3, while FS2 and FSC presented the lowest values (*p* < 0.05). The differences in permeability in the analyzed samples can be related to the defects (macrocracks) in the morphologies of the films obtained with ANN starch. An increase in permeability related to defects in morphology was also observed in films prepared using a mixture of hydroxypropyl methylcellulose/hydroxypropyl starch [18]. In addition to the presence of macrocracks, the microcracks produced by the recrystallization of the modified starch could also influence the increase in permeability [21].

### 3.5. Opacity

Transparency is a parameter that can help define the final use of food packaging since it is related to sensory perception by the customer as well as product protection. The opacity of biodegradable films is shown in Table 1. The highest opacity was observed in FS3, followed by FS1 (*p* < 0.05), while FS2 and FSC presented low opacity. A similar increase in the opacity of films obtained with HMT-modified rice starch was observed compared to those obtained with native starch [21]. The authors attributed the lower transparency of the films obtained with modified starch to conformational changes in the starch granule structure due to the reorganization by the modification process.

### 3.6. Mechanical Properties

The mechanical properties of edible films are shown in Table 1. FS2 showed the highest EB (*p* < 0.05). The higher value of EB can be related to the low relative crystallinity of the sample (Section 3.6), which indicates a lower reorganization, compared to samples FS1 and FS3, of the internal structure and, therefore, a greater elasticity. In addition, the high crystallinity decreases the interaction between the plasticizer (glycerol) and polymer chains, limiting the mobility of these chains in the amorphous region of the films due to the low intercalation of glycerol between the polymeric structure [26]. In contrast to the EB, the FSC and FS2 samples showed the lowest values of TS and YM, which is expected because the tensile strength and the modulus increase with an increase in the organization of the crystalline structure of the materials, providing greater rigidity to the films [27]. Similar behavior was observed in corn starch-based films. Generally, films with high crystallinity degrees presented high TS and YM values [28,29,30]. Another determining factor is the presence or absence of macro and microcracks in the morphology of starch annealed films which can influence the mechanical properties reducing its elasticity and others, such as barrier properties [18].

### 3.7. Attenuated Total Reflectance-Fourier Transform Infrared Spectroscopy (ATR-FTIR)

**Figure 4:**
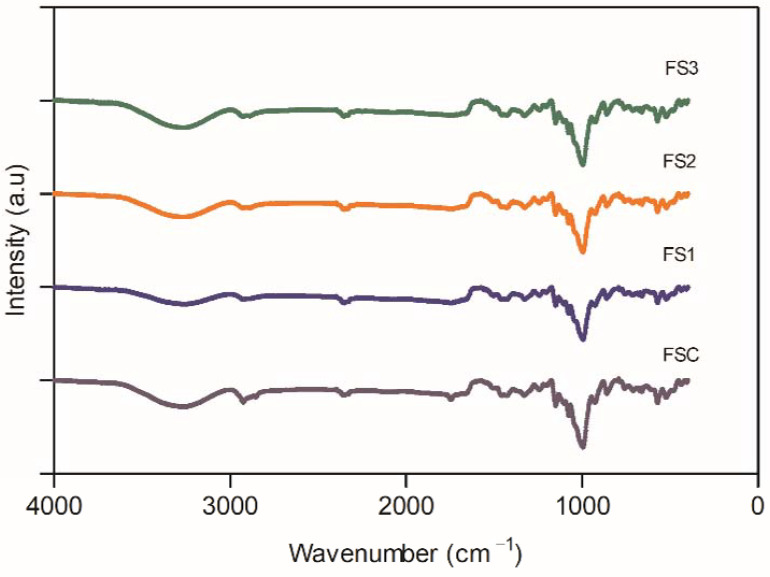
FTIR spectrums of native and modified starch-based films samples.

The ATR-FTIR spectra of samples analyzed are shown in Figure 4. All samples exhibited a band at 3300 cm^−1^ corresponding to the stretching of hydroxyl groups (–O–H) [11]. A previous report observed that the native ulluco starch and the modified starches presented the same band at 3446 cm^−1^ [7]. However, there was a slight shift in the signal in the films analyzed. This behavior was previously reported in films based on cassava starch [31]. It may be associated with the interaction between the hydroxyl groups of glycerol and native or modified starch [9]. The asymmetrical and symmetrical stretching of bonds C–H is associated with bands between 2900 cm^−1^ and 2950 cm^−1^ [32].

Additionally, bands at 1320 cm^-1^ and 1430 cm^-1^ were observed, corresponding to the bending vibration of −CH groups [31]. The bands observed at 958 cm^−1^ and 1078 cm^−1^ are related to the crystalline structure, whereas the band observed at 997 cm^-1^ is associated with the amorphous region correlated to the presence of starch in the film composition [9]. The high intensity of the signal corresponding to the amorphous region corresponds to the low relative crystallinity of the films (see Section 3.6).

### 3.8. Thermal Analysis of Starch and Edible Films

**Figure 5:**
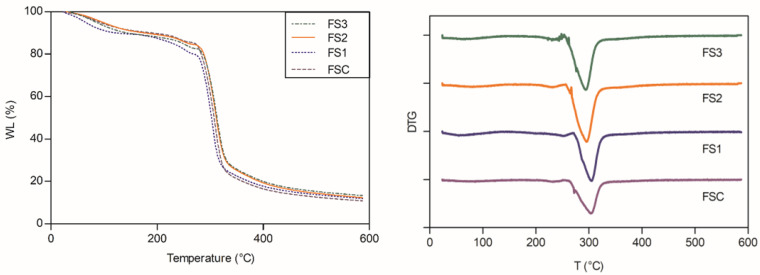
Weight loss curves of native and modified starch-based films sample.

The weight loss of films was 86.9 ± 1.9%, 88.8 ± 0.7%, 86.6 ± 1.1%, and 86.3 ± 0.5% for FSC, FS1, FS2, and FS3, respectively (*p* > 0.05). The samples showed four stages of decomposition (Figure 5): The first stage ranged between 40 °C and 150 °C, the second stage ranged between 214 °C to 269 °C, the third stage ranged between 264 °C to 324 °C, and the fourth stage occurred above 324 °C. The first stage corresponds to the water evaporation from the films, which is related to the moisture content of samples. The second stage corresponds to the degradation of the glycerol-rich phase, which is characteristic of starch films plasticized with glycerol [33]. The depolymerization of the constituents of the starch takes place in stage three, which is associated with the greatest decomposition of the analyzed samples, with weight losses between 59% and 63% at maximum decomposition temperatures between 301 °C and 304 °C. Finally, in stage four, the formation of inert carbonaceous residues occurs. No statistical differences were observed between the samples. These results agree with those reported for biodegradable films obtained using wheat, corn, and potato starch [34].

## 4. Conclusions

Ulluco starch modified by ANN showed potential as a material to produce biodegradable films. However, a significant influence of the modification process on the physicochemical characteristics of the materials evaluated, mainly in the morphology, was evidenced. In general, the modified ulluco starch improved film characteristics such as solubility and swelling power, providing greater resistance to moisture. In addition, an increase in relative crystallinity was observed, associated with a decrease in the transparency of the films. This fact may contribute to the consideration that a reduction of the transparency of the films could be related to a lower passage of light and greater protection of the food matrix. Although an increase in the crystallinity of the samples is observed, there is no evidence of a relationship between this characteristic and the barrier properties of the materials, which could be influenced by the micro and macrocracks observed on the surface of the samples. This observation suggests that more tests should be carried out to determine the optimal concentration of modified starch and plasticizer in the preparation of biodegradable films as well as the starch gelatinization time and thus obtain continuous and more consistent morphologies.

On the other hand, all the samples presented high thermal stability. However, no impact on this property was observed using modified starch. In general, ulluco starch modified by ANN offers as a viable alternative for producing biodegradable films with improved physicochemical properties. However, additional testing is required to optimize its performance.

## Figures and Tables

**Table 1:** Physicochemical properties of biodegradable films.

Sample	S (%)	SP (%)	WVP × 10^−9^ (g m^−1^ s^−1^ Pa^−1^)	Thickness	Opacity	EB (%)	YM (MPa)	TS (%)
FSC	21.8 ± 0.8 ^a^	299 ± 39 ^a^	2.18 ± 0.10 ^c^	0.13 ± 0.01	0.96 ± 0.07 ^b^	25 ± 2 ^b^	288 ± 42 ^c^	5.1 ± 0.3 ^c^
FS1	20.7 ± 0.9 ^ab^	191 ± 16 ^b^	3.45 ± 0.21 ^a^	0.11 ± 0.01	0.95 ± 0.09 ^b^	22 ± 4 ^b^	892 ± 89 ^a^	10.5 ± 0.8 ^a^
FS2	20.6 ± 0.8 ^b^	207 ± 22 ^b^	2.46 ± 0.18 ^c^	0.13 ± 0.01	1.35 ± 0.12 ^a^	30 ± 3 ^a^	237 ± 61 ^c^	5.5 ± 0.7 ^c^
FS3	19.6 ± 0.8 ^b^	153 ± 11 ^c^	2.88 ± 0.10 ^b^	0.11 ± 0.01	0.83 ± 0.04 ^c^	22 ± 3 ^b^	597 ± 77 ^b^	9.4 ± 0.5 ^b^

S: solubility; SP: swelling power; WVP: water vapor permeability; EB: elongation at break; YM: Young modulus; TS: tensile strength. Values are expressed as means ± standard deviation. Different letters in the same column indicate significant differences between samples (*p* ≤ 0.05).

## Data Availability

Data is contained within the article.

## References

[1] (2020). Plastics Europe Plastics—The Facts 2021, An Analysis of European Plastics Production, Demand and Waste Data. https://issuu.com/plasticseuropeebook/docs/plastics_the_facts-web-dec2020.

[2] Rafey A., Siddiqui F.Z. (2021). A Review of Plastic Waste Management in India–Challenges and Opportunities. Int. J. Environ. Anal. Chem..

[3] Cheng H., Chen L., McClements D.J., Yang T., Zhang Z., Ren F., Miao M., Tian Y., Jin Z. (2021). Starch-Based Biodegradable Packaging Materials: A Review of Their Preparation, Characterization and Diverse Applications in the Food Industry. Trends Food Sci. Technol..

[4] Jiang T., Duan Q., Zhu J., Liu H., Yu L. (2020). Starch-Based Biodegradable Materials: Challenges and Opportunities. Adv. Ind. Eng. Polym. Res..

[5] Bhargava N., Sharanagat V.S., Mor R.S., Kumar K. (2020). Active and Intelligent Biodegradable Packaging Films Using Food and Food Waste-Derived Bioactive Compounds: A Review. Trends Food Sci. Technol..

[6] Henning F.G., Ito V.C., Demiate I.M., Lacerda L.G. (2021). Non-conventional starches for biodegradable films: A review focussing on characterisation and recent applications in food. Carbohydr. Polym. Technol. Appl..

[7] Parra D.O., Daza Ramírez L.D., Sandoval-Aldana A., Eim V.S., Váquiro H.A. (2022). Annealing Treatment of Ulluco Starch: Effect of Moisture Content and Time on the Physicochemical Properties. J. Food Process. Preserv..

[8] Fonseca L.M., El Halal S.L.M., Dias A.R.G., Zavareze E.d.R. (2021). Physical Modification of Starch by Heat-Moisture Treatment and Annealing and Their Applications: A Review. Carbohydr. Polym..

[9] Galindez A., Daza L.D., Homez-Jara A., Eim V.S., Váquiro H.A. (2019). Characterization of Ulluco Starch and Its Potential for Use in Edible Films Prepared at Low Drying Temperature. Carbohydr. Polym..

[10] Daza L.D., Homez-Jara A., Solanilla J.F., Váquiro H.A. (2018). Effects of Temperature, Starch Concentration, and Plasticizer Concentration on the Physical Properties of Ulluco (Ullucus Tuberosus Caldas)-Based Edible Films. Int. J. Biol. Macromol..

[11] Daza L.D., Eim V.S., Váquiro H.A. (2021). Influence of Ulluco Starch Concentration on the Physicochemical Properties of Starch–Chitosan Biocomposite Films. Polymers.

[12] Valcárcel-Yamani B., Rondán-Sanabria G.G., Finardi-Filho F. (2013). The Physical, Chemical and Functional Characterization of Starches from Andean Tubers: Oca (Oxalis Tuberosa Molina), Olluco (Ullucus Tuberosus Caldas) and Mashua (Tropaeolum Tuberosum Ruiz & Pavón). Braz. J. Pharm. Sci..

[13] Feijoo P., Samaniego-Aguilar K., Sánchez-Safont E., Torres-Giner S., Lagaron J.M., Gamez-Perez J., Cabedo L. (2022). Development and Characterization of Fully Renewable and Biodegradable Polyhydroxyalkanoate Blends with Improved Thermoformability. Polymers.

[14] Baron R.D., Pérez L.L., Salcedo J.M., Córdoba L.P., Sobral P.J.d.A. (2017). Production and Characterization of Films Based on Blends of Chitosan from Blue Crab (Callinectes Sapidus) Waste and Pectin from Orange (Citrus Sinensis Osbeck) Peel. Int. J. Biol. Macromol..

[15] (2016). Standard Test Methods for Water Vapor Transmission of Materials.

[16] (2018). Standard Test Method for Tensile Properties of Thin Plastic Sheeting.

[17] Tavares K.M., de Campos A., Luchesi B.R., Resende A.A., de Oliveira J.E., Marconcini J.M. (2020). Effect of Carboxymethyl Cellulose Concentration on Mechanical and Water Vapor Barrier Properties of Corn Starch Films. Carbohydr. Polym..

[18] Chen Y., Liao L., Liu H., Wang Y., Zhang L., Chen L., Yu L. (2020). Effect of Annealing on Morphologies and Performances of Hydroxypropyl Methylcellulose/Hydroxypropyl Starch Blends. J. Appl. Polym. Sci..

[19] Mali S., Grossmann M.V.E., García M.A., Martino M.N., Zaritzky N.E. (2006). Effects of Controlled Storage on Thermal, Mechanical and Barrier Properties of Plasticized Films from Different Starch Sources. J. Food Eng..

[20] Gutiérrez T.J., Herniou-Julien C., Álvarez K., Alvarez V.A. (2018). Structural Properties and in Vitro Digestibility of Edible and pH-Sensitive Films Made from Guinea Arrowroot Starch and Wastes from Wine Manufacture. Carbohydr. Polym..

[21] Majzoobi M., Pesaran Y., Mesbahi G., Golmakani M.T., Farahnaky A. (2015). Physical Properties of Biodegradable Films from Heat-Moisture-Treated Rice Flour and Rice Starch. Starch/Staerke.

[22] Zavareze E.D.R., Pinto V.Z., Klein B., El Halal S.L.M., Elias M.C., Prentice-Hernández C., Dias A.R.G. (2012). Development of Oxidised and Heat-Moisture Treated Potato Starch Film. Food Chem..

[23] Punia Bangar S., Nehra M., Siroha A.K., Petrů M., Ilyas R.A., Devi U., Devi P. (2021). Development and Characterization of Physical Modified Pearl Millet Starch-Based Films. Foods.

[24] Shahbazi Y. (2017). The Properties of Chitosan and Gelatin Films Incorporated with Ethanolic Red Grape Seed Extract and Ziziphora Clinopodioides Essential Oil as Biodegradable Materials for Active Food Packaging. Int. J. Biol. Macromol..

[25] Alimi B.A., Workneh T.S., Femi F.A. (2021). Fabrication and Characterization of Edible Films from Acha (Digitalia Exilis) and Iburu (Digitalia Iburua) Starches. CYTA J. Food.

[26] Colivet J., Carvalho R.A. (2017). Hydrophilicity and Physicochemical Properties of Chemically Modified Cassava Starch Films. Ind. Crops Prod..

[27] Lee H., Yamaguchi K., Nagaishi T., Murai M., Kim M., Wei K., Zhang K.Q., Kim I.S. (2017). Enhancement of Mechanical Properties of Polymeric Nanofibers by Controlling Crystallization Behavior Using a Simple Freezing/Thawing Process. RSC Adv..

[28] Ibrahim M.I.J., Sapuan S.M., Zainudin E.S., Zuhri M.Y.M. (2019). Physical, Thermal, Morphological, and Tensile Properties of Cornstarch-Based Films as Affected by Different Plasticizers. Int. J. Food Prop..

[29] Osman A.F., Siah L., Alrashdi A.A., Ul-Hamid A., Ibrahim I. (2021). Improving the Tensile and Tear Properties of Thermoplastic Starch/Dolomite Biocomposite Film through Sonication Process. Polymers.

[30] Müller P., Kapin É., Fekete E. (2014). Effects of Preparation Methods on the Structure and Mechanical Properties of Wet Conditioned Starch/Montmorillonite Nanocomposite Films. Carbohydr. Polym..

[31] Mantovan J., Bersaneti G.T., Faria-Tischer P.C.S., Celligoi M.A.P.C., Mali S. (2018). Use of Microbial Levan in Edible Films Based on Cassava Starch. Food Packag. Shelf Life.

[32] Homez-Jara A., Daza L.D., Aguirre D.M., Muñoz J.A., Solanilla J.F., Váquiro H.A. (2018). Characterization of Chitosan Edible Films Obtained with Various Polymer Concentrations and Drying Temperatures. Int. J. Biol. Macromol..

[33] Sessini V., Arrieta M.P., Kenny J.M., Peponi L. (2016). Processing of Edible Films Based on Nanoreinforced Gelatinized Starch. Polym. Degrad. Stab..

[34] Basiak E., Lenart A., Debeaufort F. (2017). Effect of Starch Type on the Physico-Chemical Properties of Edible Films. Int. J. Biol. Macromol..

